# An elemental diet is effective in the management of diversion colitis 

**Published:** 2021

**Authors:** Andrew Lane, Nicholas Dalkie, Lisa Henderson, James Irwin, Kamran Rostami

**Affiliations:** 1 *Department of Gastroenterology, Palmerston North Hospital, New Zealand*; 2 *Lisa Henderson,Dietetic/Nutrition, Palmerston North Hospital, New Zealand*

**Keywords:** Elemental diet, Diversion colitis, Crohn’s disease, Ileostomy

## Abstract

The use of an elemental diet in the management of inflammatory gastroenterological diseases has long been accepted as standard management and has shown to both induce and maintain remission in Crohn’s disease, but the evidence is lacking for its use in diversion colitis. An elemental diet is one which provides all the required nutrition in a more easily absorbed and hypoallergenic form. In this case report, we present a patient who had a flare of diversion colitis treated with diet alone. She had a significant improvement in her symptoms with a decrease in bowel motions, rectal discharge and pain. This case suggests that there may be some role for the use of an elemental diet in the management of diversion colitis. We also examine some potential mechanisms which may lead to the benefit observed.

## Introduction

 The use of diet has long been widely accepted as an important part of standard management in gastroenterological disorders, and an exclusive elemental diet has been shown to both induce and maintain remission in patients with Crohn’s disease (1,2). In contrast, evidence is lacking for the use of diet, namely an elemental diet and the LOFFLEX diet, in the management of diversion colitis (3).

Diversion colitis as an entity was first described in 1974 by Morson and colleagues (4) and has subsequently been described and examined in multiple studies. It is thought that at 3-36 months post-surgery more than 90% of patients develop some degree of diversion colitis endoscopically but only a small proportion of these patients (30%) become symptomatic (3,5). Diversion colitis is defined by inflammation of a section of bowel that has been isolated from functioning bowel after surgery, such as an ileostomy or colostomy formation (3,6). The most effective management of these patients is surgical reversal of the stoma and regeneration of intestinal continuity. Medical therapy however, is still a key component of disease control in both those preparing for surgery and those for whom surgical intervention is not a viable option, either due to sepsis, peri-anal disease, incontinence or technical difficulty (3). These medical therapies include interventions such as corticosteroids, 5-aminosaliccylic acid (5-ASA), and short-chain-fatty acids (SCFA), but as previously mentioned, there is no evidence for the use of diet and lifestyle changes in the management of patients with diversion colitis (3), even with an understanding that nutritional imbalances play a key role in the pathology of diversion colitis.

An exclusive elemental diet involves providing patients with essential and non-essential amino acids, fats, carbohydrates and vitamins in a form that is more effectively absorbed and less allergenic in patients with gastrointestinal disorders (1). While the LOFFLEX diet has been designed to help re-introduce foods after enteral feeding, it consists of a low-fibre, fat-limited exclusion diet that includes those foods reported to cause the least symptoms in patients with inflammatory bowel disease (7). The LOFFLEX diet consists of an initial two-week phase in which the basic diet is followed, followed by a reintroduction phase in which new foods are slowly added while monitoring symptoms (7). 

 The aim of the current case report is to discuss the potential role of an exclusive elemental diet in the management of diversion colitis. 

## Case Report

A 29-year-old woman with ileocolonic Crohn’s disease diagnosed fifteen years ago (2004), for whom a right hemi-colectomy with loop ileostomy was performed twelve years ago. A reversal of the ileostomy was attempted but failed and she had to be reversed back to an ileostomy. She was subsequently medically managed with a number of treatments including corticosteroids, 6-mercaptopurine, Adalimumab and most recently she was managed with Ustekinumab with variable effectiveness. Socially she lived with her husband and child and was a smoker of 10/day and drank alcohol occasionally.

In 2019, she presented with two weeks of increased rectal discharge of mucus and blood 10-15 times a day with some abdominal pain and bloating despite being on Ustekinumab. The ileostomy was still functioning normally. On examination, her ileostomy bag was in situ, her abdomen was soft without any significant tenderness on palpation.

**Figure 1:**
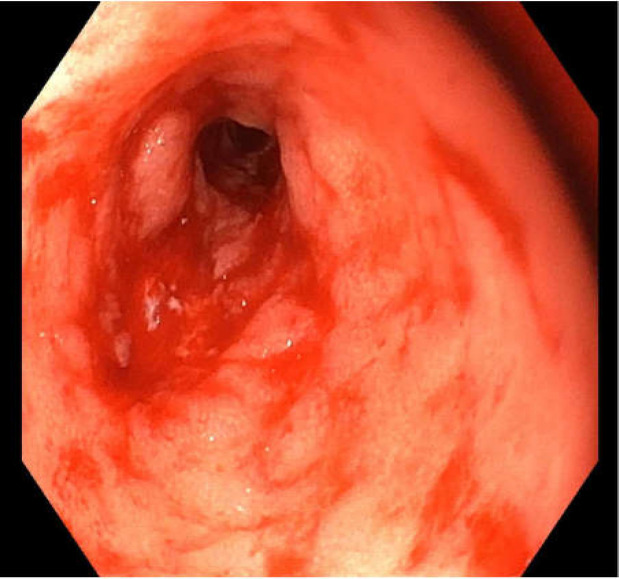
Stenotic inflamed segment in the diverted colon with loss of mucosal folds and vascular markings demonstrated at sigmoidoscopy

**Figure 2:**
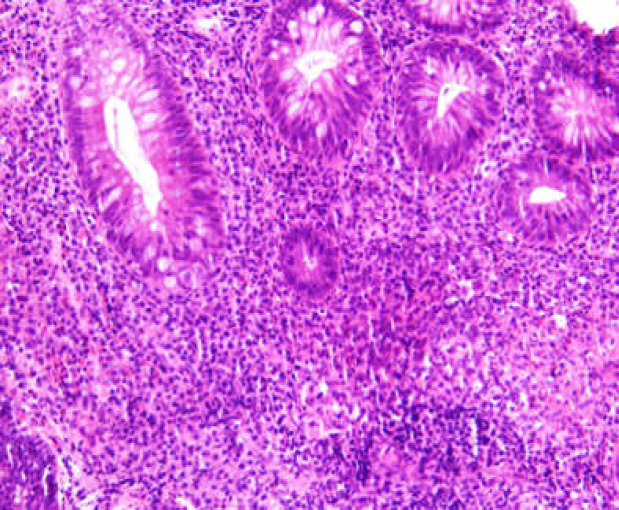
Recto-sigmoid biopsy taking from the diverted colon showing Neutrophils extending into the glands consistent with an acute colitis

Investigations revealed a C-reactive protein (CRP) of 7.4 and a recent fecal calprotectin from her ileostomy was in the normal range with a calprotectin from her rectal discharge of <1000ug/g. Her haemoglobin was 142g/L and white blood cell count of 7.2x10^9^/L. A flexible sigmoidoscopy performed one month after the onset of symptom showed an absence of mucosal folds or vascular markings with friability causing marked contact bleeding from the scope (Figure 1). There was a stricture noted at 15cm that could not be passed. Histology from a colon biopsy demonstrated features consistent with acute colitis with neutrophils extending into the glands and focal areas of mucosal ulceration (Figure 2). 

She was introduced to dietary management of Crohn’s disease and started an exclusive Elemental 028 diet (table 1) followed by the LOFFLEX diet under the guidance and instructions of a dietitian. She was subsequently prescribed topical corticosteroids after her sigmoidoscopy, but declined to take it due to previous adverse reactions. Therefore, she was treated solely with the dietary interventions of an exclusive elemental diet follow by the LOFFLEX diet.

During her follow-up clinic review, she noted initial difficultly with the diet but had a significant improvement in symptoms with improvement in rectal discharge with no blood or mucus, and her bowel motions improved from 10-15 times a day to 1-3 times a day without further blood or mucus. After this improvement, it was decided to stop her Ustekinumab as it did not seem to have any role in controlling her symptoms.

**Table 1:** Composition of the Elemental 028 enteral feed (8)

Energy (kJ/100g)	1614
Protein (g/100g)/source	13.8/Synthetic
Carbohydrate(g/100g)/source	64.5/glucose and sucrose
Total fat (g/100g)	17.3
Total long-chain(g/100g)/source	11.2/Safflower and Canola
Total medium-chain	0
Saturated fat (% total)	12.5
Monounsaturated fat (% total)	66
Polyunsaturated fat (% total)	21.5

## Discussion

The role of dietary intervention in diversion colitis has not been studied and seems to be underestimated. It is unclear whether this modality has any important impact in the treatment of diversion colitis. We report a patient with diversion colitis who experienced a full symptoms’ resolution following treatment with an exclusive elemental diet and the LOFFLEX diet.

Despite numerous studies into the mechanisms driving diversion colitis, the pathogenesis is still unclear, but there are a number of theories that guide management options. Glotzer et al. (9) proposed that it may be as a result of bacterial overgrowth in the dysfunctioning bowel leading to an overgrowth of harmful bacteria and a disequilibrium between the luminal flora and the gut mucosa (9). It thus causes more production of nitric oxide which becomes toxic within the colon mucosa at high amounts (3). The treatment is then autologous fecal microbiota transplantation (FMT), which has proven safe and effective for a number of gastrointestinal disorders, including diversion colitis (10). Since FMT plays a a role in the management of diversion colitis, elemental diets are being recognized for their ability to support the re-balancing of gut flora. 

Other thoughts around the pathogenesis of diversion colitis suggest that ischemia in the isolated bowel plays a role related to nutritional deficiencies provided by the fecal stream (11). This stems from the benefit that is obtained from the treatment with SCFA enema and its role in the relaxation of vascular smooth muscle and decreasing tone in the pelvic arteries. SCFAs are usually produced by colonic bacteria during the breakdown of carbohydrates and thus provide an energy source of the colonic mucosa (12). Alternative treatments with 5-ASA and topical corticosteroid have been shown to be effective and recommended in symptomatic patients and for whom surgical restoration of bowel continuity is pending or not an option (3). Despite evidence suggesting the role of nutrition in the pathogenesis of diversion colitis, there is no current evidence to support the use of diet (3), namely an elemental diet, in the treatment of diversion colitis.

An elemental diet consists of a liquid diet that contains all the required protein and nutrients in their digested forms and has been shown to be as effective as corticosteroids in the treatment of inflammatory bowel conditions such as Crohn’s disease (1). The low-fibre nature of the diet also allows for the treatment of gut inflammation (13) and small bowel bacterial overgrowth (1). It is reported that the low antigenic properties of an elemental diet can help reduce the immune stimuli in the gut and allow for gut rest (13).

The patient in the current case report was treated with diet alone, namely an exclusive elemental diet followed by the LOFFLEX diet. It was shown to have significant benefit in symptoms related to a flare of diversion colitis. Her pain significantly improved and her bowel motions decreased from 15 times a day to just 1-2 times. She also had complete resolution of the discharge of blood and mucus from the isolated bowel. There are potentially multiple mechanisms by which an elemental diet could lead to an improvement in symptoms, but we hypothesize that an elemental diet plays a role in clearing the antigens from the bowel in the form of complex proteins and carbohydrates. This in turn lowers autoimmunity and inflammation in the diverted colon. Use of an elemental diet supports this theory in that decreasing antigen load in the gut may help to regulate autoimmunity which can cause expanded diversion colitis. 

Another evidence is reported by Podas et al. (14) who examined the use of an elemental diet versus oral corticosteroids in the treatment of rheumatoid arthritis. This study showed that two weeks of an elemental diet was as effective as corticosteroids in improving subjective clinical parameters in rheumatoid arthritis. The authors suggest that the decreased antigen load leads to a decreased intestinal permeability and suppression of gut mucosal associated lymphoid tissue (MALT), reducing migration of lymphocytes to the synovium (12). Thus, a similar mechanism may be contributing to the benefit that was seen in the current case in the diverted colon after a few weeks of an elemental diet.

Diversion colitis is a common complication of ileostomy or colostomy formation and the underlying pathogenesis is still not fully understood. Although surgical reversal is still considered the gold standard treatment for these patients with symptomatic diversion colitis, medical therapy still plays a key role in management. This case has highlighted that an elemental diet may be a safe and effective treatment for these patients and may reduce side effects associated with other lines of therapy such as corticosteroids. Further research is needed to inspect both the efficacy and potential mechanisms of an elemental diet in the management of diversion colitis.
